# Shear capacity of corrugated web steel beams strengthened with CFRP strips

**DOI:** 10.1038/s41598-024-76347-4

**Published:** 2024-12-16

**Authors:** Mohamed A. EL Aghoury, Alshaymaa K. Dyab, Sherif M. Ibrahim, Amr B. Saddek

**Affiliations:** 1 Structural Engineering Department, Faculty of Engineering, Ain Shams University, Cairo, Egypt; 2 Structural Engineering Department, Faculty of Engineering, Beni-Suef University, Beni-Suef, Egypt

**Keywords:** Strengthening, Shear, Carbon fiber reinforced polymer (CFRP), Corrugated web, Steel, Engineering, Civil engineering

## Abstract

In recent years, there has been significant advancement in strengthening techniques for steel structures using carbon-fiber reinforced polymer (CFRP). While numerous studies have focused on CFRP strengthening of steel beams with flat webs, similar investigations on corrugated web steel beams (CWSBs) remain limited despite their increasing application in various steel structures. This study presents numerical and analytical investigations aimed at evaluating the effectiveness of CFRP strengthening for CWSBs and developing a design procedure to predict the shear buckling capacity of strengthened CWSBs. The research employs a finite element (FE) model developed using ANSYS software, validated against previous experimental work by the same authors, which accurately reflects the overall behavior of CWSBs. A parametric study is conducted on 105 CWSBs using the validated FE model to assess the impact of various geometrical parameters, including beam web slenderness ratio, length and thickness of CFRP strips, and different CFRP strip schemes. Results demonstrate that CFRP strengthening can enhance the shear capacity of CWSBs by up to 74.50%. The study identifies the arrangement of CFRP strips on both sides of the web as the most effective parameter for controlling the efficiency of the CFRP strengthening technique. Conversely, changes in CFRP strips up to 70% of web height have minimal effect. A proposed design procedure for predicting the design shear buckling strength of CFRP-strengthened CWSBs shows good consistency with FE model results.

## Introduction

Many existing steel structures are experiencing a reduction in load-carrying capacity due to increased load demands resulting from upgraded usage and function. Consequently, there is a growing need to adopt suitable strengthening techniques for these structures to withstand the additional loads. One promising method involves the use of advanced composite materials, such as Carbon Fiber Reinforced Polymer (CFRP). CFRP composites possess excellent properties, including high strength-to-weight ratio, durability, corrosion resistance, flexibility in shaping, ease of construction, and ease of maintenance, making them superior to conventional retrofitting methods^1,2^.

Several studies have investigated the shear and buckling strengthening of flat web steel beams reinforced with CFRP^3–10^. These studies reported that CFRP strengthening techniques enhance the shear capacity of steel beams. Zhao^11^ and Sayed-Ahmed^4^ explored the use of CFRP to increase the buckling capacity of beams by applying CFRP strips to the web, demonstrating that CFRP can delay or reduce web buckling, thereby increasing shear capacity by enhancing the web’s buckling capacity. The slenderness ratio significantly affects the efficiency of CFRP strengthening. Zhao and Al-Mahaidi^6^ studied the effects of CFRP strengthening on the web-buckling capacity of light-steel beams by applying CFRP laminates to one or both sides of the web. Their test results indicated that CFRP strengthening significantly increases web-buckling capacity, especially for beams with high web slenderness ratios and for beams bonded on both web sides.

Patnaik et al.^7^ experimentally studied the effects of CFRP strengthening on beam shear strength by reinforcing two beams with CFRP strips attached to the web. The results showed an increase in shear capacity of up to 26%. Narmashiri et al.^8^ examined the impact of applying CFRP on steel webs with different configurations and arrangements. They found that CFRP reinforcement increased the shear capacity and decreased the deformation of steel beams. Using two and three CFRP strips, the shear capacity increased by 51.41 and 51.67%, respectively, when applied to both web sides, and by 35.39% and 43.48%, respectively, when applied to one web side. Narmashiri^9^ also studied the effect of different CFRP strip orientations (vertical and diagonal) and arrangements (one-side or both-sides) on beam shear capacity, finding that applying CFRP strips on both web sides increased shear capacity by 25% for vertical CFRP and 34% for diagonal CFRP compared to unstrengthened beams. The increase in shear capacity was higher for beams with CFRP strips on both web sides compared to those with strips on one side, by 9% for vertical CFRP and 14% for diagonal CFRP.

Nhut and Matsumoto^10^ investigated the effects of CFRP strengthening on the shear strength of thin-walled steel plates (TSPs) by applying CFRP strips with three different fiber orientations (0°, 90°, and 45°). The results indicated an increase in the shear strength of TSPs, and high-accuracy theoretical equations were proposed to calculate shear strength. Teng et al.^1^ noted that the bond strength of FRP-to-steel bonded joints is the maximum tensile force resisted by the composite repair before debonding occurs, governed by interfacial behavior along the surface interaction. Fracture mechanics (energy-based methods) are used to predict bond strength, with cohesive behavior between two separating faces defined by a traction-separation law (TSL) based on the bond-slip relationship of the CFRP/steel interface, introducing the failure mechanism and yielding critical inter-laminar fracture energy.

Corrugated web steel beams (CWSBs) have been used instead of classical steel beams with stiffened flat webs due to their superior advantages, such as enabling the use of thinner webs and providing higher load capacity at lower costs^12^. The elastic local shear buckling stress of CWSBs can be determined by classical plate buckling theory^13^. Elastic global shear buckling strength for CWSBs has been provided by^14,15^ by treating the corrugated web as an orthotropic flat web. Various researchers have developed different analytical models to estimate interactive shear stress^16–20^. Barakat and Leblouba^21^ experimentally and analytically investigated the shear strength of CWSBs and developed a model to estimate normalized interactive shear buckling strength. Wang et al.^22^ proposed a new calculation method for predicting the elastic critical shear buckling stress of large-scale CWSBs in bridge girders, improving the prediction accuracy of previous formulas by considering the dimension differences of corrugated steel plates in buildings and bridge girders. They reported that previous methods overestimate interactive shear buckling stress values for small web heights and underestimate them for relatively larger web heights.

The current study aims to predict the shear capacity of CWSBs strengthened with CFRP strips. To achieve this, a research program was conducted by the authors at Ain Shams University, including both experimental and numerical phases. The experimental study, published by Dyab et al.^23^, involved tests on five CWSBs with a length of 1512 mm, including one unstrengthened specimen and four specimens strengthened with different CFRP strip arrangements and schemes. The results showed an increase in shear capacity of 25–69% for the CFRP-strengthened CWSBs.

The current research phase focuses on developing a numerical model to accurately predict the shear capacity of CWSBs strengthened with CFRP strips. The numerical investigation was conducted using a finite element (FE) model developed with ANSYS software, and the results were validated against the experimental data^23^. A parametric study on 105 CWSB models was conducted using the validated FE model to investigate the influence of different parameters, including the slenderness ratio of both web and fold, fold aspect ratio, CFRP strip length to web height ratio, CFRP strip thickness, and CFRP schemes and arrangements. A proposed design procedure was developed to predict the design shear buckling strength of CWSBs strengthened with CFRP.

## Nonlinear finite element analyses

Three-dimensional non-linear finite element model of CWSBs strengthened by CFRP strips was created using finite element package ANSYS 19 software^24^ to simulate the tested specimens. The FE model results are validated against the experimental ones presented in Dyab et al.^23^.

### Description and geometry of the model

**Fig. 1:**
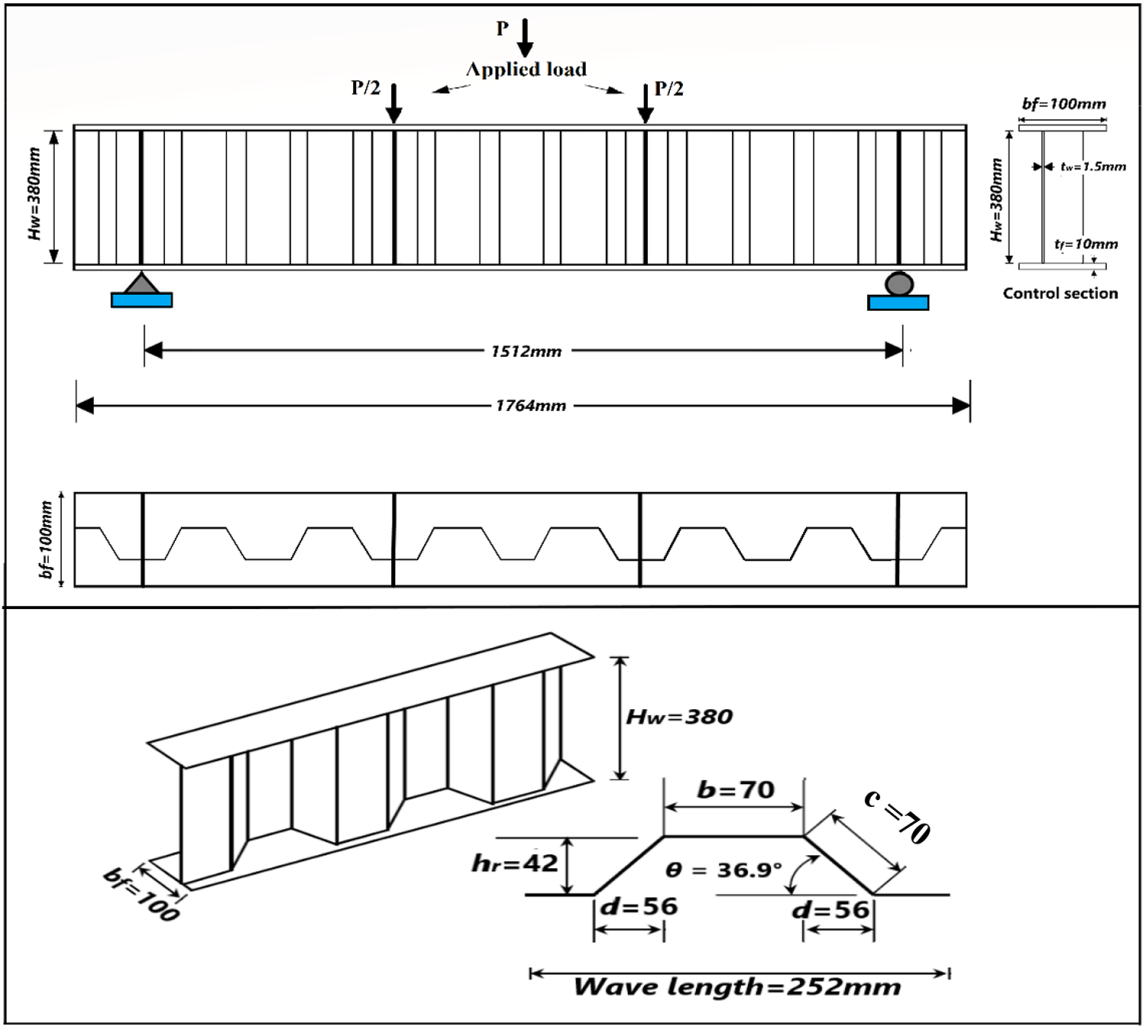
Steel corrugated web beam’s dimensions^23^.

**Fig. 2:**
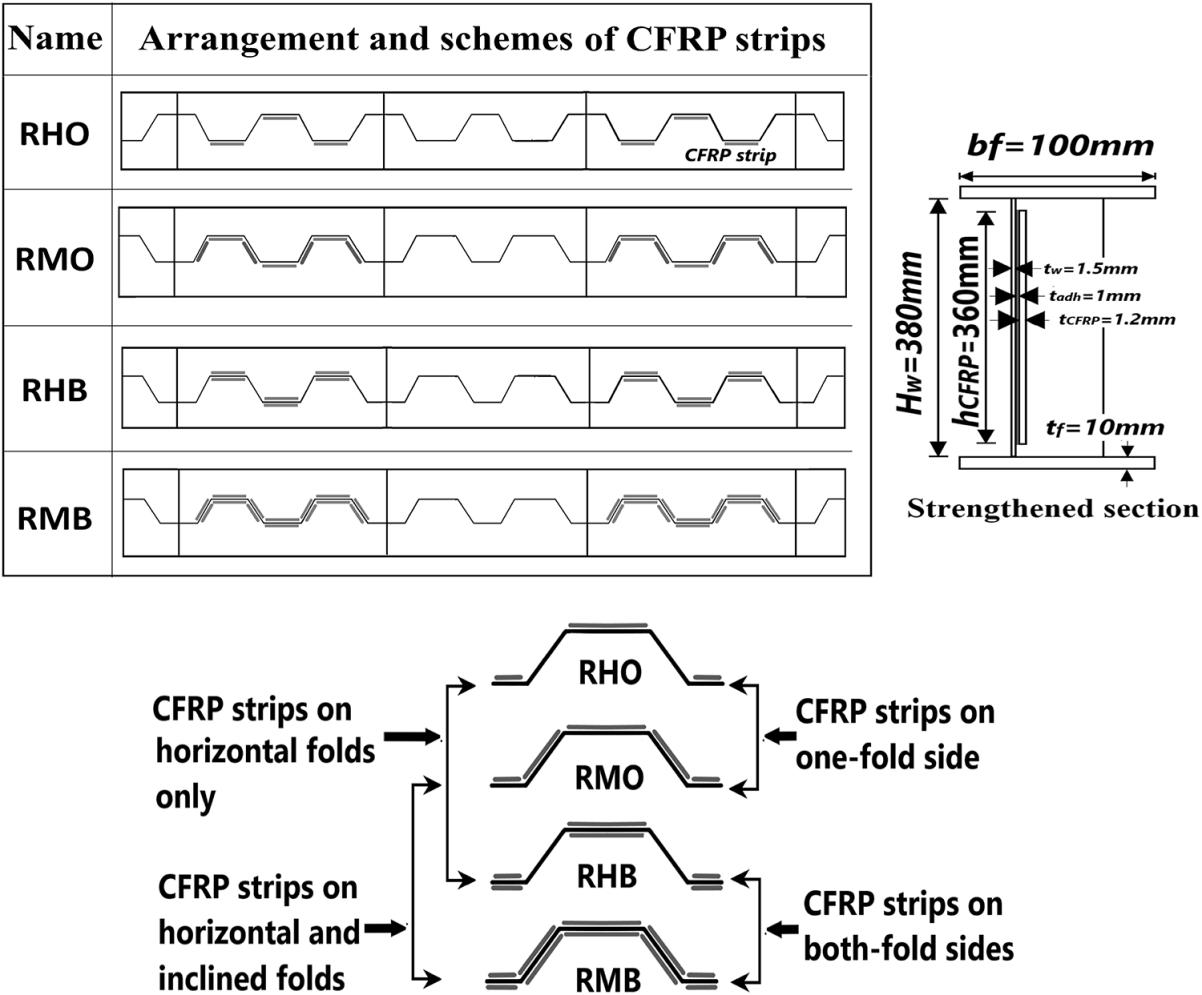
CFRP strengthening configuration^23^.

The dimensions, cross-sections, strengthening configuration and the boundary conditions of the tested strengthened CWSBs^23^ shown in Fig. 1; and Table 1 were used to perform numerical modelling. The overall configuration of CFRP strengthened test specimens and strengthened section dimensions are shown in Fig. 2 while unstrengthened beam which represents the control beam is denoted (RC).

**Table 1:** Steel corrugated web beam’s dimensions^23^.

Beam dimensions (mm)						Web corrugation dimensions (mm)			
Web height “*H*_*w*_*”*	Web thickness “*t*_*w*_”	Flange width “*b*_*f*_”	Flange thickness “*t*_*f*_”	Beam length “*L*”	Shear panel width “*L*_s_”	Flat fold width “*b*”	H. projection of inclined fold “*d*”	Corrugation height “*h*_*r*_”	Corrugation angle “θ”
380	1.5	100	10	1512	504	70	56	42	36.9°

**Fig. 3:**
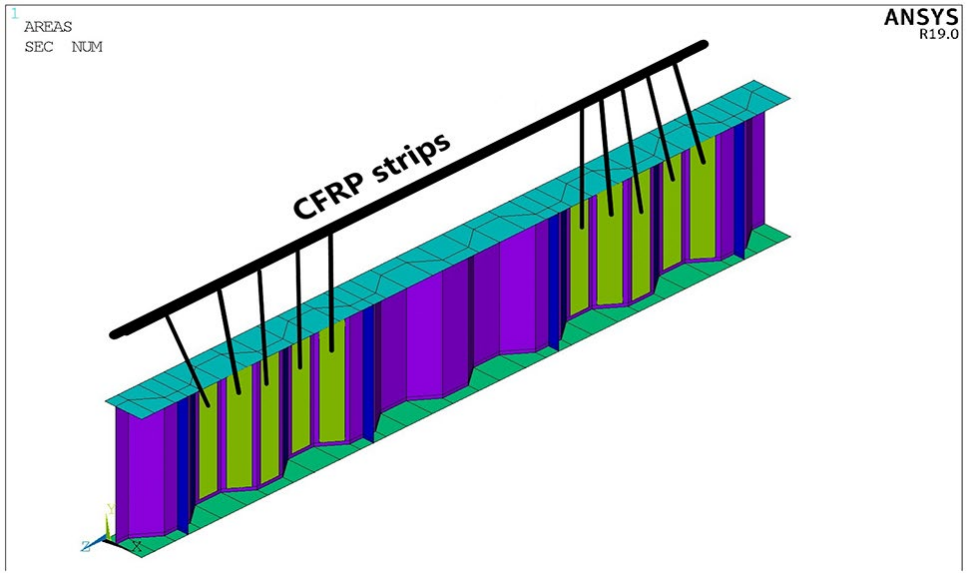
Strengthened beam with horizontal and inclined CFRP strips.

**Fig. 4:**
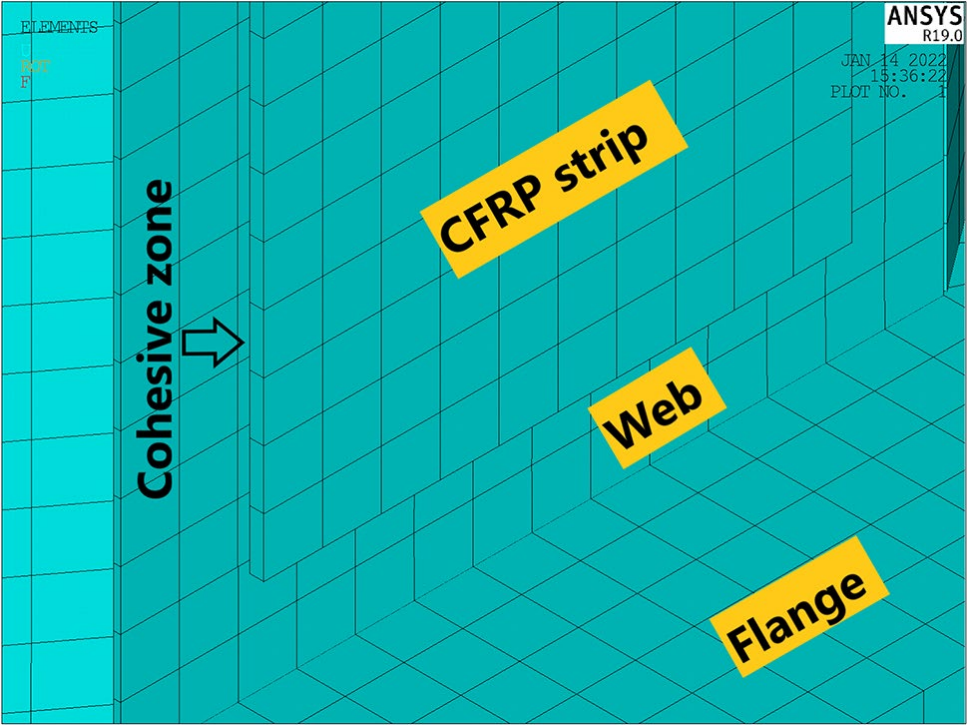
The adhesive modelling.

A model of strengthened CWSB with CFRP strips on horizontal and inclined folds is shown in Fig. 3. The adhesive is modeled physically by offsetting the CFRP strips at a distance of 1 mm from the steel web as shown in Fig. 4. As this space represents the cohesive zone distance which will be simulated by cohesive zone material (CZM) model, which discussed later in Sect. 2.5.

### Loading and boundary conditions

**Fig. 5:**
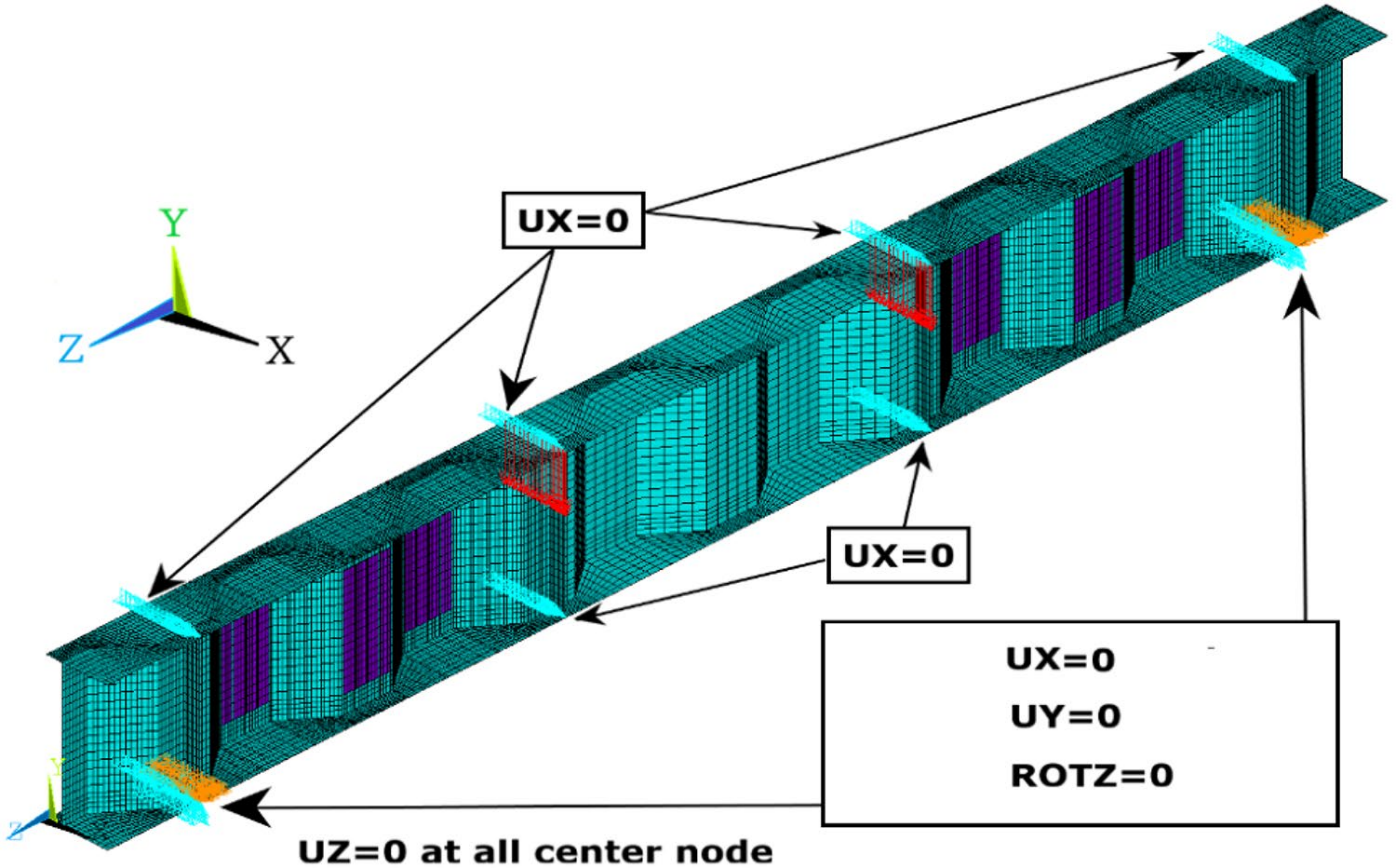
Loading system and boundary conditions.

The two end sections are simply supported where for all nodes of each support section, the twist rotation about longitudinal direction (z-axis) and the vertical displacement along (y-axis) are restrained. Also, the displacement in longitudinal direction (z-axis) at a center point of one support is restrained to maintain the stability of CWSBs. To avoid any torsional deformation, the beams were laterally supported in out-of-plan (x-axis) at all nodes of each support section and all nodes of the top flange section at loading points. The loading system and boundary conditions are shown in Fig. 5.

### Material properties

The mechanical properties of the steel specimens as reported by Dyab et al.^23^ are shown in Table 2.

**Table 2:** Steel mechanical properties^23^.

Part / strength	Yield (MPa)	Ultimate (MPa)
Flanges	384	502
Web	260	365

The CFRP strips of pultruded Sika^®^ Carbodur^®^ plate S512 type, epoxy resin of Sikadur^®^ 30 type provided by SIKA^®^ CO., Swiss branch, are used. The used CFRP is factory-pultruded plate consists of unidirectional, stretched carbon fibers in resin matrix. The mechanical properties of CFRP plates and adhesive are taken as given in manufacturer data sheet, these properties and dimensions summarized in Tables 3, 4 respectively.

**Table 3:** CFRP strip’s dimensions and properties.

Sika^®^ Carbodur^®^ plate (S512)								
Dimensions (mm)		E-modulus (N/mm^2^)						
Width	Thickness	Mean value		Min. value	5% Fracture value		95% Fracture value	
50	1.2	165,000		> 160,000	162,000		180,000	
Tensile strength (N/mm^2^)					Strain			
Mean value	Min. value	5% Fracture value		95% Fracture value	Strain at break		Design strain	
3100	> 2800	3000		3600	> 1.7%		< 0.85%	

**Table 4:** Adhesive’s dimensions and properties.

Sikadur^®^ -30					
Dimensions (mm)		Compressive strength (N/mm²)		Tensile strength (N/mm²)	
Width	Thickness	E-modulus	Strength at 7 days	E-modulus	Strength at 7 days
50	1	9,600	70–95	11,200	24–31
Shear strength (N/mm²)				Bond strength on steel (N/mm²)	
Strength at 7 days				Mean value	Min. value
14–19				> 30	> 21

**Fig. 6:**
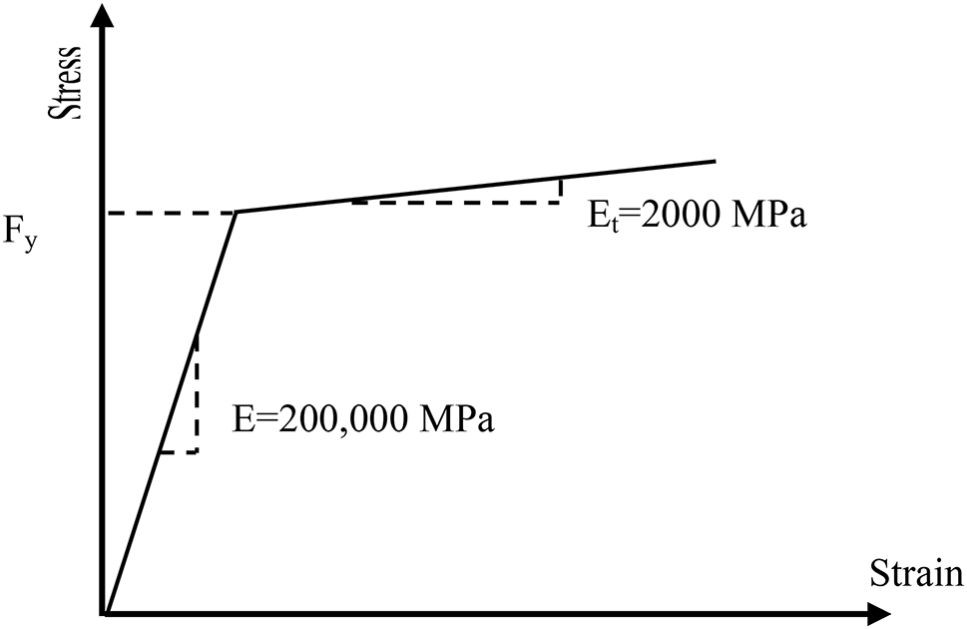
Steel stress-strain curve.

The steel material of CWSB is modeled as a bilinear stress–strain curve with linear strain hardening; plastic tangent modulus (E_t_) was assumed as (E/100) as shown in Fig. 6. The material of the CFRP strips is unidirectional which is defined as linear and orthotropic.

### Elements and meshing

The FE model is built using shell elements considering a fine mesh. The element meshed with a ratio not exceeding 1:2. For zones occupied with CFRP strips, a fine mesh is used with the element size fixed to 5 mm. Both steel beam and CFRP strip are modeled using (Shell-181) with reduced integration. Shell-181 has 4-node and has six degrees of freedom at each node. The shell elements modelling both steel and CFRP are separated by the adhesive thickness, and they were connected through contact pair that is explained in the following section.

### Interfacial surface interaction behavior of contact pair

**Fig. 7:**
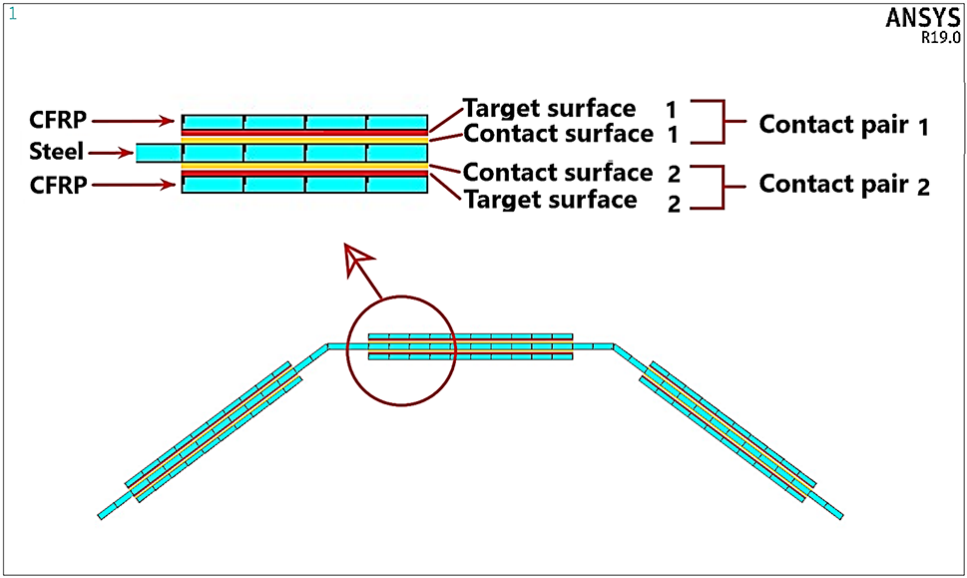
The created contact pairs.

**Fig. 8:**
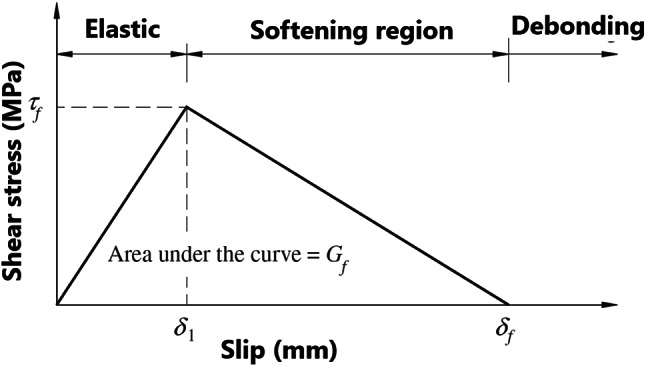
A bi-linear bond-slip model^28^.

The bond strength of the adhesive joint is governed by interfacial behavior along the interface surface interaction between contact pair surfaces (Steel & CFRP surfaces), (see Fig. 7). To model this interfacial behavior of contact pair surfaces and predict the CFRP debonding process, cohesive zone material (CZM) model approach available in the ANSYS^®^ Mechanical™ software is used. In CZM approach, a model for the CFRP/steel interface is used to introduce failure mechanisms by using the relationship between interfacial stress (Traction) and the relative slip/ separation at interface. This relationship is illustrated by a bond-slip/separation model. Debonding propagation may be introduced in three different failure modes and loads, denoted I (separation mode), II (slip mode) and III (mixed mode)^25^. For mode I, bond-separation model is used, and an approximation model using the tensile stress-strain data of the adhesive material can be used^26^. Campilho et al.^26^ assumed that the peak stress of the bond-separation model can be taken the same as the tensile strength of the adhesive and the separation at complete failure can be taken as the product of the tensile strain at complete failure and the adhesive thickness. For mode II, bond-slip model is used and the model which was proposed based on research study of Xia S and Teng JG^27^ can be used. The bond-slip model was further developed by Fernando^28^ based on his experimental results of bonded joints done for adhesives with linear and non-linear elastic adhesives. For linear adhesive, Fernando^28^ proposed a bi-linear shear stress-slip relationship (see Fig. 8), while for non-linear adhesive he proposed a trapezoidal stress-slip relationship. As the type of adhesive used in the current research project is linear adhesive (SKIADUR-30) the bi-linear shear stress-slip model in^28^ is adopted in this study. The key parameters of this bi-linear bond-slip model are calculated from mechanical properties of the joint adhesive using the following equations provided by Fernando^28^:

1 $$\:\:{G}_{f}=628\:{{t}_{a}}^{0.5\:}{R}^{2}$$

2 $$\:{\tau\:}_{max}=0.9\:{\sigma\:}_{max}$$

3 $$\:{\delta\:}_{1}=0.3{\left(\frac{{t}_{a}}{{G}_{a}}\right)}^{0.65}\:{\sigma\:}_{max}$$

4 $$\:{\delta\:}_{f}={\left(\frac{2{G}_{f}}{{\tau\:}_{max}}\right)}^{.}$$

Where:

$\:{G}_{f}$, is the critical interfacial fracture energy for tangential slip, (MPa)

$\:{\tau\:}_{max}$, is maximum equivalent tangential contact stress, (MPa)

$\:{\delta\:}_{1}$, is the slippage of CFRP at the maximum equivalent tangential contact stress, (mm)

$\:{\delta\:}_{f}$, is the slippage of CFRP at the completion of debonding, (mm)

t_a_, is the adhesive thickness in (*mm*),

R, is the tensile strain energy of the adhesive; $\:R={\left(\frac{{{\sigma\:}^{2}}_{max}}{2{E}_{a}}\right)}^{.}$ in (*MPa*),

σ_max_, Tensile strength of the adhesive in (*MPa*),

G_a_, is the shear modulus of the adhesive in (*MPa*).

E_a_ is the elastic modulus of the adhesive in (*MPa*).

In the FE model contact pair elements (Contact/Target) are used to model the interface delamination. The contact element which supports debonding in ANSYS^®^ Mechanical™ software is surface-to-surface contact element (CONTA173) associated with a target elements TARGET 170 with a shared real constant^24^. The debonding for a contact pair is activated by specifying a CZM model. The contact element (CONTA173) has the capability to include both mode (I), bond-separation and mode (II) bond slip. The recommended bilinear CZM model which follows a bilinear traction-separation law is used and defined using the data table method (TB and TBDATA commands) using two different ways of defining the input material data. The two ways are tractions-separation distances or tractions - critical fracture energies. The used input material data belongs Mode (I) are calculated from the tensile stress-strain data of the adhesive material. These data are the maximum normal contact stress (The tensile strength of the adhesive), the critical interfacial fracture energy for normal separation and the separation of CFRP at the completion of debonding. The used input material data belonging to Mode (II) is calculated from bond-slip model using adopted Eqs. (1)–(4) provided by Fernando^28^. These data are the maximum equivalent tangential contact stress, the critical interfacial fracture energy for tangential slip and the slippage of CFRP at the completion of debonding.

The material constants parameters (C1 through C6) used for contact element (CONTA173) on the TBDATA command in ANSYS are assigned for bond-separation & bond-slip relationships, as follows:

- The first two parameters (C1 and C2) belong to Mode I which is calculated from the tensile stress-strain data of the adhesive material, which are define as follow:
- The maximum normal contact stress (The tensile strength of the adhesive) is (C1),
- The separation of CFRP at the completion of debonding is (C2) which can be calculated as the product of the tensile strain at complete failure and the adhesive thickness.
- The second two parameters (C3 and C4) belong to Mode II which calculated from bond-slip model using adopted Eqs. (1, 2, 4), which are defined as follow:
- The maximum equivalent tangential contact stress, τ_max_, is (C3),
- The critical interfacial fracture energy for tangential slip, $\:{G}_{f}$, is (C4 in CBDE) and
- The slippage of CFRP at the completion of debonding, $\:{\delta\:}_{f}$, is (C4 in CBDD).
- The parameter (C5) is an artificial damping coefficient (η) which is used to stabilize the numerical solution for convergence issue in the Newton-Raphson solution and program default value is used.
- The parameter (C6) is a control of tangential slip under compressive normal contact stress for mixed mode debonding and program default value is used.

### Nonlinearity and initial imperfection

Large deformation is considered by adjusted ANSYS command (NLGEOM). Large deformation is considered by updating the element orientation which influences element stiffness in each deformation stage. The solution is performed as a nonlinear-static analysis by employing the ANSYS Newton-Raphson technique to solve the nonlinear equilibrium equations by a displacement-controlled mode and dividing the applied loads into a series of load steps and sub-steps. Material nonlinearity is considered in steel material which is modeled as a bilinear stress–strain curve with linear strain hardening, as shown in Fig. 6. However, the material of the CFRP strips defined as linear material and the adhesive material is modelled with a cohesion zone material (CZM) model as discussed in previous section. In this research study, linear buckling analysis is run first to determine the buckling shape and use it for implementing the initial imperfection. The initial geometrical imperfection is assigned in the non-linear analysis by scaling the first buckling mode shape from linear buckling analysis to value of out-of-plane imperfections with a magnitude of h_w_/200 as recommended by Eurocode 3^29^.

### Verification of FE model against experimental results

**Fig. 9:**
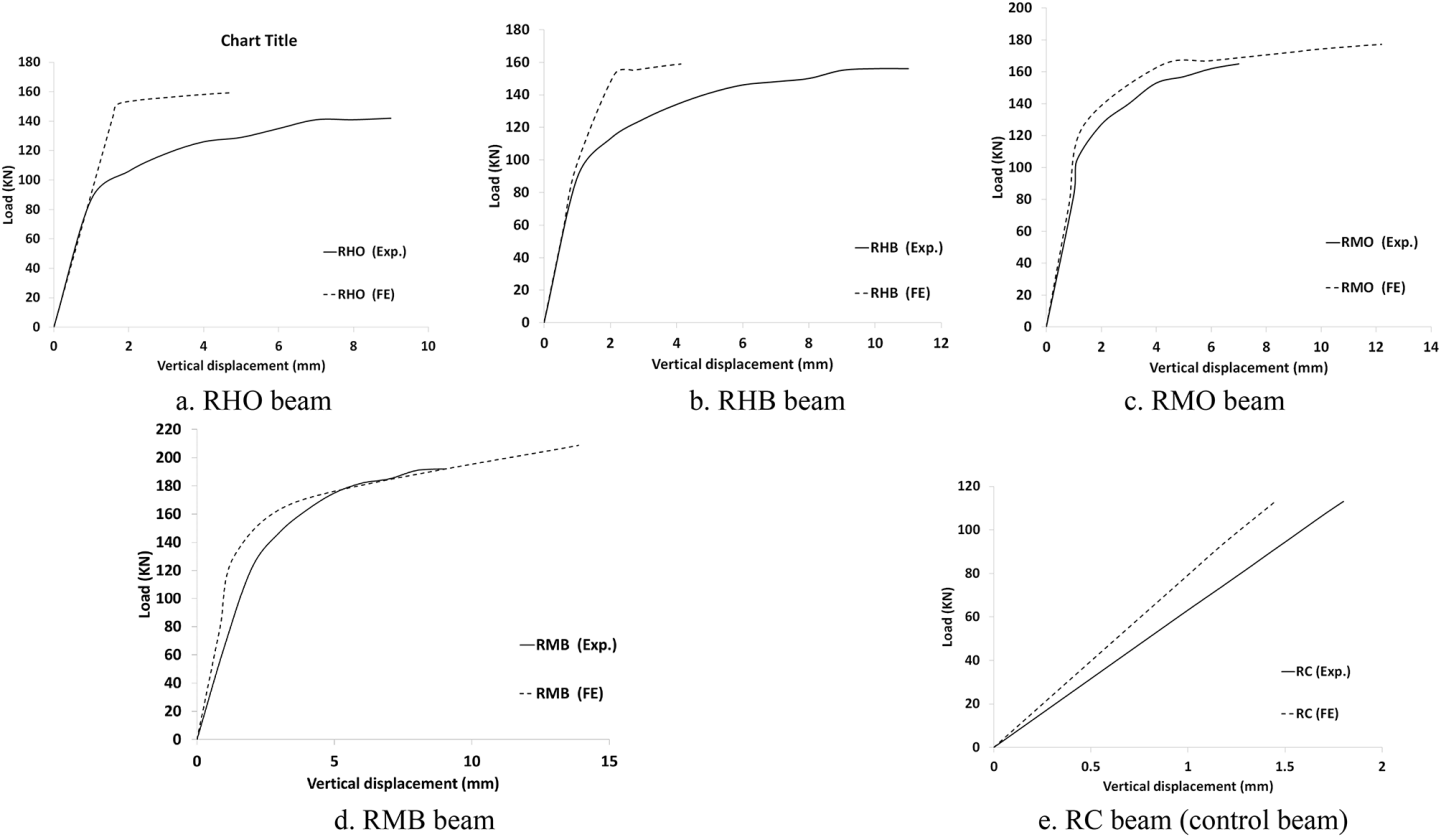
Load- displacement curve for RC beams and each strengthened beam (Experimental vs. FE).

**Fig. 10:**
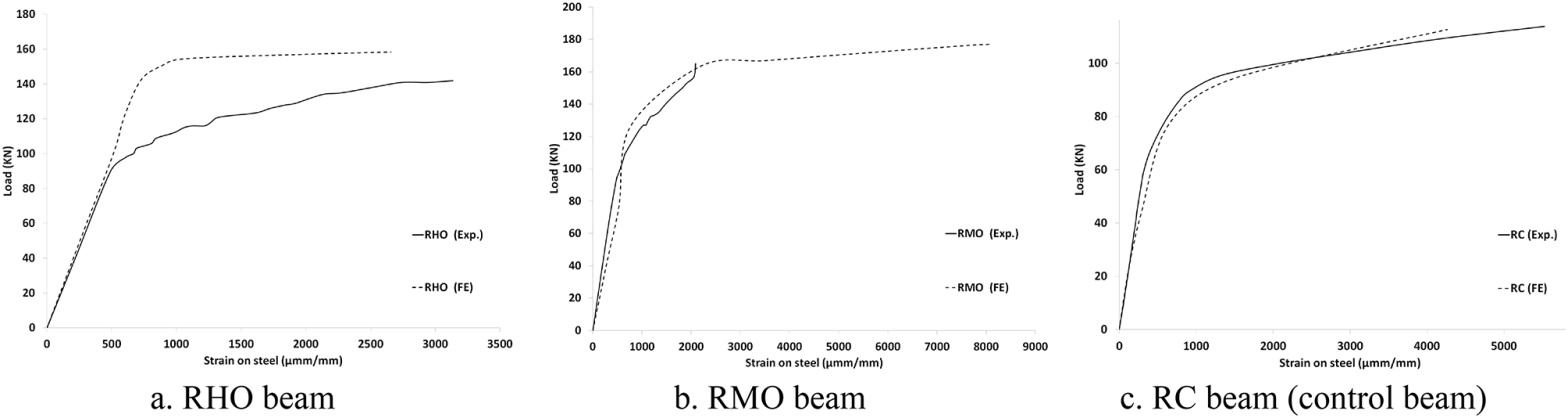
Load- strain curve for RC beams against each strengthened beam except RHB, RMB (Experimental vs. FE).

**Fig. 11:**
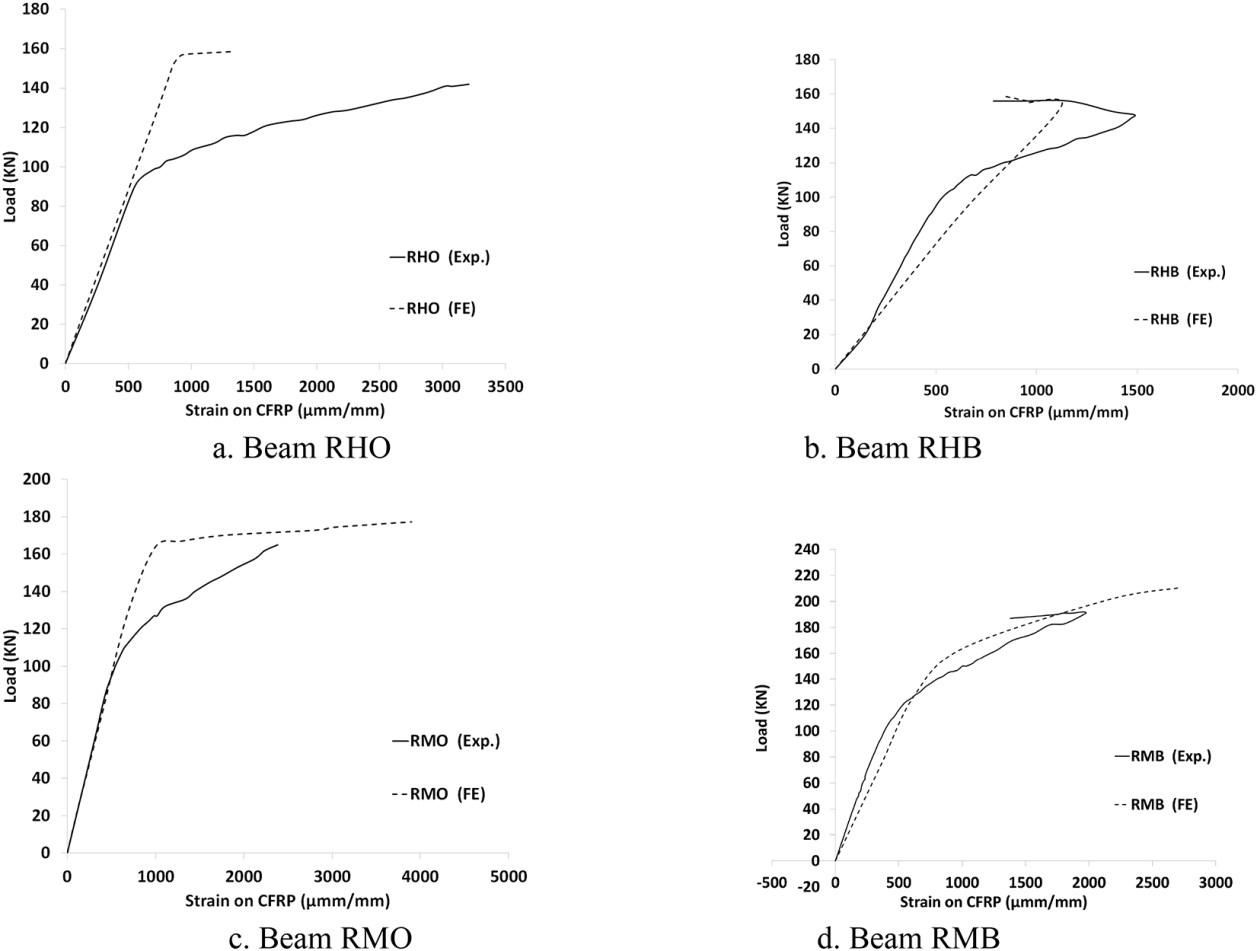
Load- CFRP strain curve for each strengthened beam (Experimental vs. FE).

**Fig. 12:**
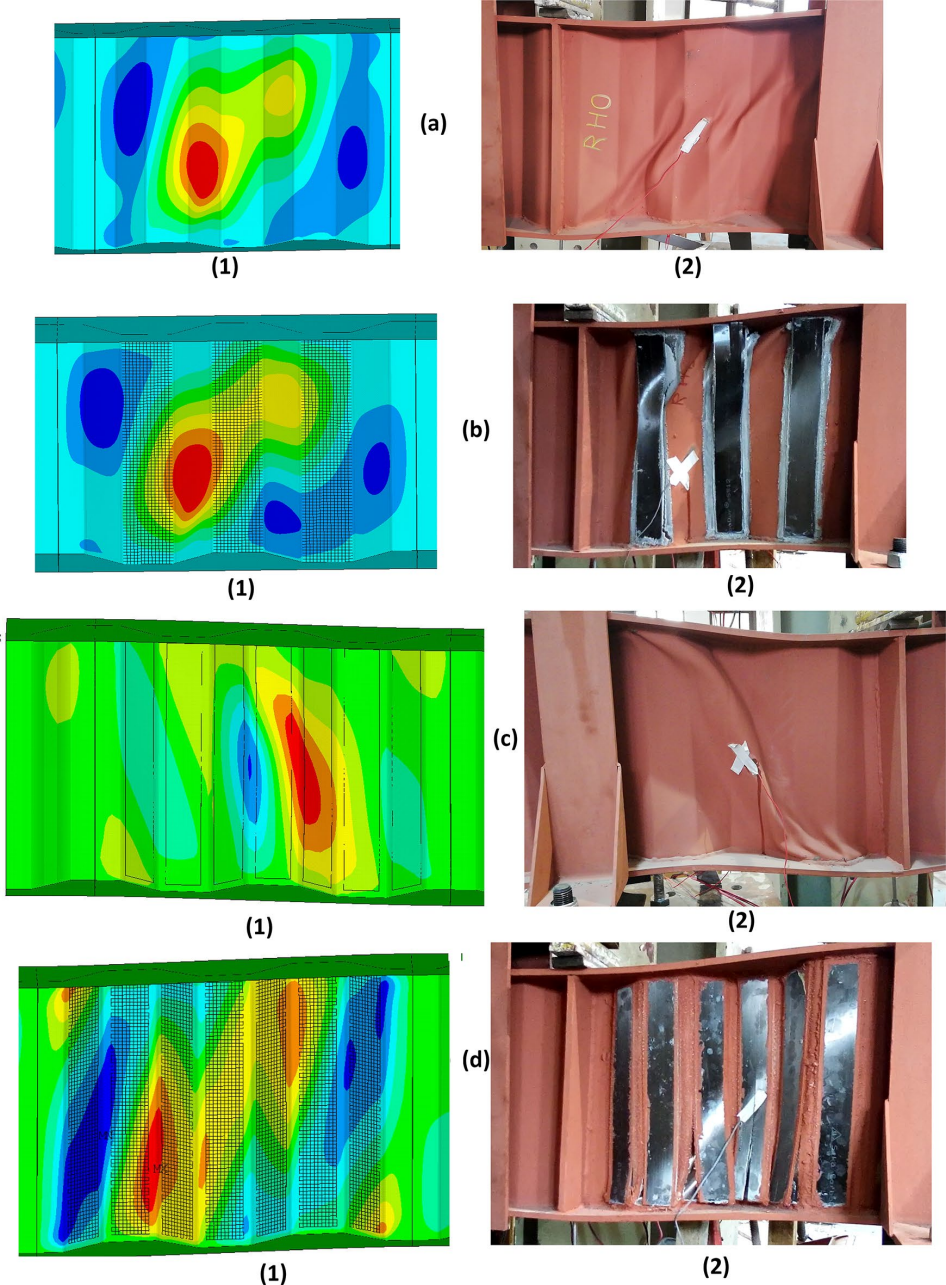
Comparison of failure shapes of tested beams and FE model. (**a**). Failure of beam RHO. (**b**). Failure of beam RHB. (**c**). Failure of beam RMO. (**d**). Failure of beam RMB. (1) Numerical model. (2) Experimental beam^23^.

The results of the FE model are compared to the experimental of Dyab et al.^23^. Table 5 indicates that the FE models accurately predicted the ultimate load capacity of the CWSBs. The load-displacement, load- strain and load-CFRP strain relationship extracted from the FEA are plotted against the corresponding experimental readings^23^, as shown in Figs. 9, 10, 11 respectively which indicate that the FE simulation results were consistent with the test results. A comparison between the failure shapes obtained in FE models and those observed in the experimental investigation indicates the accuracy of the proposed FE model to simulate the actual behavior of CWSBs as shown in Fig. 12.

Table 5 indicates that the variation between the ultimate load predicted by the finite element (FE) model and the experimental results ranges from 1.92 to 11.27%. Overall, the FE model tends to overestimate the ultimate load in comparison to the experimental test results, with an average overestimation of 6.82% and a standard deviation of 3.3%. The discrepancies between the FE model predictions and the experimental results can be attributed to several factors:

- Differences in the actual imperfection shape and magnitude of the experimental specimens compared to those assumed in the FE model,
- Non-uniformity in the thickness of the adhesive layer between the steel surface and CFRP strips in the experimental specimens, which may influence bond strength, particularly in relation to the bond-separation model compared to the FE model, and.
- Variations in the actual mechanical properties of the adhesive due to the curing process in the experimental specimens, as opposed to the material properties used in the FE model based on data from the material supplier.

**Table 5:** A comparison between the results of experimental and FE ultimate loads.

Beam name	Experimental ultimate load^23^ (kN)	FE ultimate load (kN)	Error percentage of ultimate load in FE relative to Exp. (%)
RC	114	119	4.39
RHO	142	158	11.27
RHB	156	159	1.92
RMO	165	177	7.27
RMB	192	210	9.38

## Parametric study

The different parameters that likely affect the efficiency of strengthened CWSBs are studied in this section by performing a parametric study using the validated FE model.

### Parametric study program

The parameters which are considered in this parametric study are summarized as follows:

1. Effect of slenderness of the web (H_w_/t_w_); the studied values are (200, 300, 400)
2. Effect of slenderness of the Fold (b/t_w_); the studied values are (11.67, 47.50, 103.33)
3. Effect of aspect ratio of the Fold (b/H_w_); the studied values are (0.06, 0.16, 0.26)
4. Effect of CFRP strip length to web height (L_CFRP_/H_w_); three values are taken (90%,80%,70%)
5. The effect of thickness of CFRP strips (t_CFRP_); two values are taken (1.2 mm ,1.4 mm)
6. Effect of CFRP schemes; H (CFRP on horizontal folds), M (CFRP on all folds)
7. Effect of CFRP arrangement; O (CFRP on one-fold side), B (CFRP on both-fold sides)

Parametric study involves 105 CWSBs divided into three main groups according to web height (H_w_); (1200 mm, 800 mm and 500 mm). The first main group is divided into three sub-main groups according to beam corrugation dimensions which are assigned according to the expected buckling mode of CWSBs: global (G), interactive (I) and Local (L). Each sub-main group is divided into two groups according to CFRP strip thickness (t_CFRP_); (1.2 mm ,1.4 mm), then divided into three sub-groups according to CFRP strip length to web height (L_CFRP_/H_w_); (90%,80%,70%). Finally, each sub-group includes the four configurations of strengthened CWSBs. Each main group of the second and third main groups is divided into three groups according to web slenderness ratios: 200, 300, and 400. Each group includes only the four configurations of strengthened CWSBs, with a constant CFRP thickness (t_CFRP_=1.2) and constant CFRP strip length to web height (L_CFRP_/H_w_=90%).

Table 6 presents CWSB dimensions for all groups. The details of each main group are presented in Tables 7, 8, 9, 10 , 11.

**Table 6:** Steel corrugated web beam’s dimensions.

Main group name	Sub-main group name	Web height	Flange width	Flange thickness	Web thickness	Longitudinal fold width	Corrugation angle	Corrugation height
		*H*_*w*_	*b*_*f*_	*t*_*f*_	*t*_*w*_	*b*	*θ*	*h*_*r*_
H1200	G1200	1200	400	40	6.00	70	37	42
	I1200	1200	300	30	4.00	190	37	114
	L1200	1200	300	30	3.00	310	37	186
H800	H800-200	800	400	30	4.00	70	37	42
	H800-300	800	400	30	2.67	70	37	42
	H800-400	800	400	30	2.00	70	37	42
H500	H500-200	500	300	20	2.50	70	37	42
	H500-300	500	300	20	1.67	70	37	42
	H500-400	500	300	20	1.25	70	37	42

Main group name	Sub-main group name	Horizontal projection of Inclined fold	Web slenderness ratio	Folder slenderness ratio	Fold aspect ratio	Wave number	Beam length	S hear panel width
		*d*	*H*_*w*_ */t*_*w*_	*b/t*_*w*_	*b/H*_*w*_	*W.N.*	*L*	*L*_*s*_
H1200	G1200	56	200	11.67	0.06	30	7560	2520
	I1200	152	300	47.50	0.16	12	8208	2736
	L1200	248	400	103.333	0.26	9	10,044	3348
H800	H800-200	56	200	17.50	0.09	18	4536	1512
	H800-300	56	300	26.22	0.09	18	4536	1512
	H800-400	56	400	35.000	0.09	18	4536	1512
H500	H500-200	56	200	28.00	0.14	12	3024	1008
	H500-300	56	300	42.00	0.14	12	3024	1008
	H500-400	56	400	56.00	0.14	12	3024	1008

**Table 7:** Details of first sub-main group (Global).

Main group	Sub-main group	Group	Sub-group	CFRP schemes
Web height	Buckling mode corrugation	CFRP strips thickness t_CFRP_	CFRP strip length to web height L_CFRP_/H_w_	Model name
H1200	G1200	–		G1200 control
		1.2	90%	G1200 (N90-HO)
				G1200 (N90-HB)
				G1200 (N90-MO)
				G1200 (N90-MB)
			80%	G1200 (N80-HO)
				G1200 (N80-HB)
				G1200 (N80-MO)
				G1200 (N80-MB)
			70%	G1200 (N70-HO)
				G1200 (N70-HB)
				G1200 (N70-MO)
				G1200 (N70-MB)
		1.4	90%	G1200 (K90-HO)
				G1200 (K90-HB)
				G1200 (K90-MO)
				G1200 (K90-MB)
			80%	G1200 (K80-HO)
				G1200 (K80-HB)
				G1200 (K80-MO)
				G1200 (K80-MB)
			70%	G1200 (K70-HO)
				G1200 (K70-HB)
				G1200 (K70-MO)
				G1200 (K70-MB)
First letter	First digits	Second letter	Second two digits	Latest two letters
“G” stand for global buckling mode corrugation	Stand for web height	Stand for CFRP strip thickness; N(1.2 mm), K(1.4 mm)	Stand for the ratio of CFRP strip length to web height; (90%,80%,70%)	Stand for CFRP configuration; H (on horizontal folds), M (on horizontal and inclined folds), O (on one-fold side), B (on both-fold sides)

**Table 8:** Details of first sub-main group (interactive).

Main group	Sub-main group	Group	Sub-group	CFRP schemes
Web height	Buckling mode corrugation	CFRP strips thickness t_CFRP_	CFRP strip length to web height L_CFRP_/H_w_	Model name
H1200	I1200	–		I1200 control
		1.2	90%	I1200 (N90-HO)
				I1200 (N90-HB)
				I1200 (N90-MO)
				I1200 (N90-MB)
			80%	I1200 (N80-HO)
				I1200 (N80-HB)
				I1200 (N80-MO)
				I1200 (N80-MB)
			70%	I1200 (N70-HO)
				I1200 (N70-HB)
				I1200 (N70-MO)
				I1200 (N70-MB)
		1.4	90%	I1200 (K90-HO)
				I1200 (K90-HB)
				I1200 (K90-MO)
				I1200 (K90-MB)
			80%	I1200 (K80-HO)
				I1200 (K80-HB)
				I1200 (K80-MO)
				I1200 (K80-MB)
			70%	I1200 (K70-HO)
				I1200 (K70-HB)
				I1200 (K70-MO)
				I1200 (K70-MB)
First letter	First digits	Second letter	Second two digits	Latest two letters
“I” stand for interactive buckling mode corrugation	Stand for web height	Stand for CFRP strip thickness; N(1.2 mm), K(1.4 mm)	Stand for the ratio of CFRP strip length to web height; (90%, 80%,70%)	Stand for CFRP configuration; H (on horizontal folds), M (on horizontal and inclined folds), O (on one-fold side), B (on both-fold sides)

**Table 9:** Details of first sub-main group (local).

Main group	Sub-main group	Group	Sub-group	CFRP schemes
Web height	Buckling mode corrugation	CFRP strips thickness t_CFRP_	CFRP strip length to web height L_CFRP_/H_w_	Model name
H1200	L1200	–		L1200 control
		1.2	90%	L1200 (N90-HO)
				L1200 (N90-HB)
				L1200 (N90-MO)
				L1200 (N90-MB)
			80%	L1200 (N80-HO)
				L1200 (N80-HB)
				L1200 (N80-MO)
				L1200 (N80-MB)
			70%	L1200 (N70-HO)
				L1200 (N70-HB)
				L1200 (N70-MO)
				L1200 (N70-MB)
		1.4	90%	L1200 (K90-HO)
				L1200 (K90-HB)
				L1200 (K90-MO)
				L1200 (K90-MB)
			80%	L1200 (K80-HO)
				L1200 (K80-HB)
				L1200 (K80-MO)
				L1200 (K80-MB)
			70%	L1200 (K70-HO)
				L1200 (K70-HB)
				L1200 (K70-MO)
				L1200 (K70-MB)
First letter	First digits	Second letter	Second two digits	Latest two letters
“L” stand for local buckling mode corrugation	Stand for web height	Stand for CFRP strip thickness; N(1.2 mm), K(1.4 mm)	Stand for the ratio of CFRP strip length to web height; (90%,80%,70%)	Stand for CFRP configuration; H (on horizontal folds), M (on horizontal and inclined folds), O (on one-fold side), B (on both-fold sides)

**Table 10:** Details of second main group (H800).

Main group	Group	Group	Sub-group	CFRP schemes
Web height	Slenderness ratio	CFRP strips thickness t_CFRP_	CFRP strip length to web height L_CFRP_/H_w_	Model name
H 800	H800-200	–		H800-200 control
		1.2	90%	H800-200 (HO)
				H800-200 (HB)
				H800-200 (MO)
				H800-200 (MB)
	H800-300	–		H800-300 control
		1.2	90%	H800-300 (HO)
				H800-300 (HB)
				H800-300 (MO)
				H800-300 (MB)
	H800-400	–		H800-400 control
		1.2	90%	H800-400 (HO)
				H800-400 (HB)
				H800-400 (MO)
				H800-400 (MB)
First letter and digits		Second digits	Second two letters	
Stand for web height		Stand for slenderness ratio	Stand for CFRP configuration; H (on horizontal folds), M (on horizontal and inclined folds), O (on one-fold side), B (on both-fold sides)	

**Table 11:** Details of third main group (H500).

Main group	Group	Group	Sub-group	CFRP schemes
Web height	Slenderness ratio	CFRP strips thickness t_CFRP_	CFRP strip length to web height L_CFRP_/H_w_	Model name
H 500	H500-200	–		H500-200 control
		1.2	90%	H500-200 (HO)
				H500-200 (HB)
				H500-200 (MO)
				H500-200 (MB)
	H500-300	–		H500-300 control
		1.2	90%	H500-300 (HO)
				H500-300 (HB)
				H500-300 (MO)
				H500-300 (MB)
	H500-400	–		H500-400 control
		1.2	90%	H500-400 (HO)
				H500-400 (HB)
				H500-400 (MO)
				H500-400 (MB)
First letter and digits		Second digits	Second two letters	
Stand for web height		Stand for slenderness ratio	Stand for CFRP configuration; H (on horizontal folds), M (on horizontal and inclined folds), O (on one-fold side), B (on both-fold sides)	

In this parametric study, the material properties of CFRP strips and adhesive were considered similar to that of the specimens used in the verification part. However, ASTM A36 Steel was used as the material of construction of the CWSBs with material properties indicated in Table 12. The boundary conditions of CWSBs were modeled as simply supported beams with lateral supports to top flanges at supports and the intermediate loading points.

**Table 12:** Mechanical properties of A36 steel.

Ultimate tensile strength, MPa	540
Yield strength, MPa	250
Elongation, %	20
Modulus of elasticity (Young’s modulus), GPa	200
Shear modulus, GPa	79.3
Bulk modulus, GPa	140
Poisson’s ratio	0.26

### Results

The effects of different parameters on CWSB strength have been presented using bar charts which clearly reveal how sensitive parameters have the major effect on strengthening efficiency.

#### Effect of buckling mode on strength

**Fig. 13:**
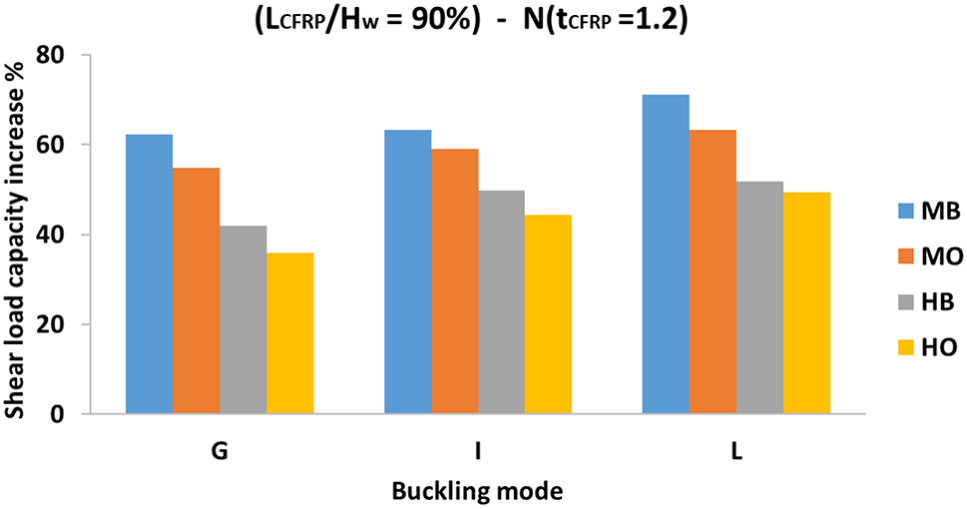
The effect of buckling mode for L_CFRP_/H_w_ = 90%, t_CFRP_ =1.2.

The effect of buckling mode is considered in the three sub-main groups; (G1200, I1200, L1200). It is clear from the illustrated bar charts in Fig. 13, which present the effect of web buckling mode on strength, that the CFRP strengthening has the greatest effect on the beams with a local buckling mode (L), compared to the beams with a global buckling corrugation (G). This effect is strongly noticed in specimens with CFRP on horizontal folds only and placed one side of the web (HO configuration), for instance, specimen L1200 (N90-HO) demonstrated a 48.9% increase in shear load capacity, whereas specimen G1200 (N90-HO) showed a comparatively lower increase of 35.8% which is almost 73.2% of that for specimen L1200 (N90-HO). Specimen I1200 (N90-HO) exhibited an intermediate increase in shear load capacity, with a value of 44.1%.

#### Effect of CFRP strip length to web height on strength

The effect of CFRP strip length to web height is considered only in the first main group (H1200).

**Fig. 14:**
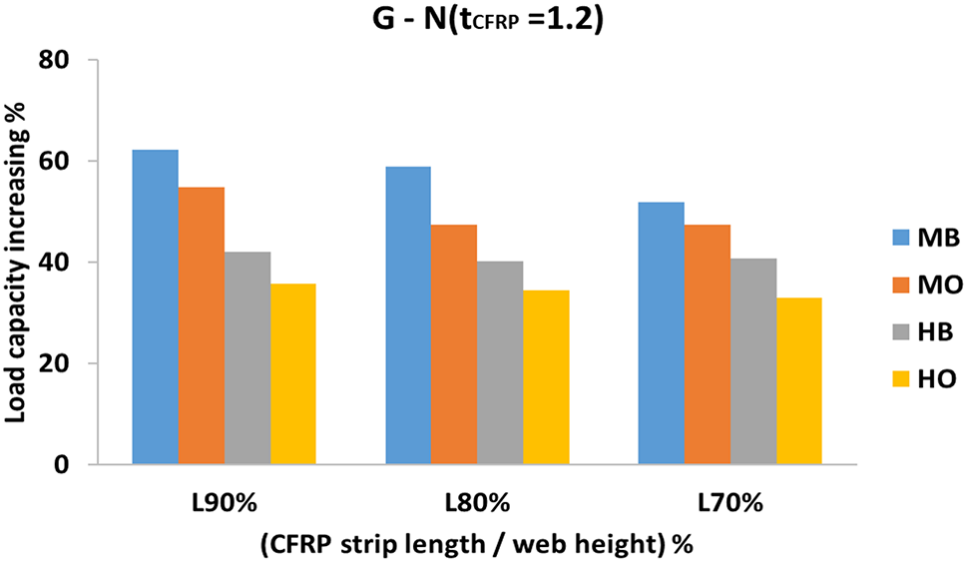
The effect of CFRP strip length to web height on strength for global buckling mode corrugation (G), t_CFRP_ =1.2.

From the illustrated bar charts in Fig. 14, for all web slenderness ratios, the increase in the length of the CFRP did not result in a significant increasing in beam capacity, as the (L_CFRP_ / H_w_) = 70% has already covered the part exposed to the maximum diagonal compressive stress resulting from the shear force in the web. For example, specimen G1200 (N90-HO) demonstrated a 35.8% increase in shear load capacity. In comparison, similar specimens with different CFRP length ratios, G1200 (N80-HO) and G1200 (N70-HO), exhibited slightly lower increases of 34.7 and 33.3%, respectively. This suggests that the 70% CFRP length ratio achieved approximately 93% of the shear load capacity increase observed with the 90% CFRP length ratio.

#### The effect of thickness of CFRP strips on strength

**Fig. 15:**
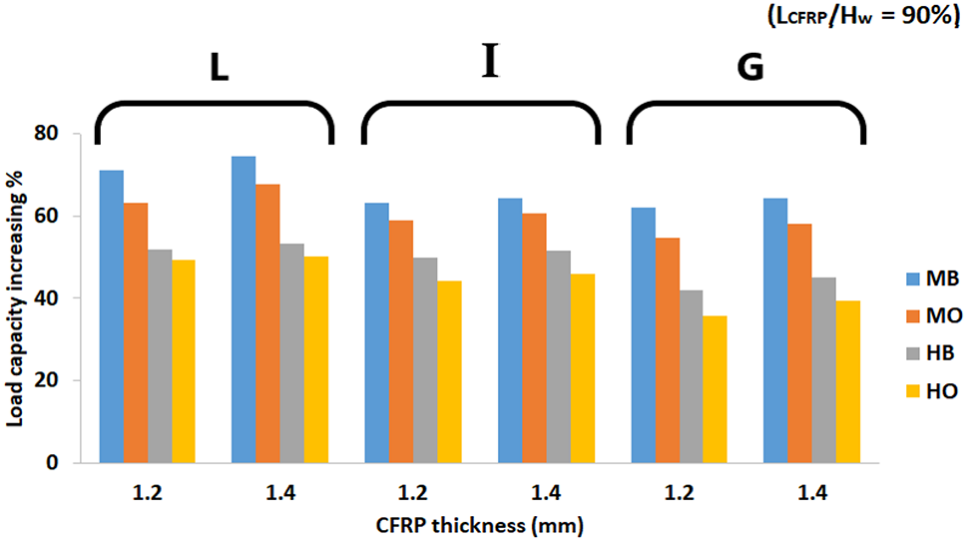
The effect of CFRP thickness for L_CFRP_/H_w_ = 90%.

Figure 15 clearly shows that the strength gains have a slight increase with the increase in CFRP thickness. The load carrying capacity is generally governed by the local buckling which depends on web thickness. Although strengthened the web with CFRP strips means increasing in the effective thickness in web, However, the change of CFRP thickness from 1.2 to 1.4 mm has insignificant effect on strength gains. Examining two specimens from the local buckling sub-group, L1200 (N90-MB) with a CFRP thickness of 1.2 mm and L1200 (K90-MB) with a CFRP thickness of 1.4 mm, it was observed that their increases in shear load capacity were 71.2 and 74.1%, respectively, indicating a difference of only 4%.

#### Effect of different CFRP configurations on strength

The effect of applying CFRP with different configurations on strength have been observed in all previous bar charts which clearly show that, among all CFRP configurations, applying CFRP on horizontal and inclined folds on both fold-sides has the largest strength gain increasing. The maximum increase in shear load capacity was observed in the first sub-group, specifically in specimen L1200 (K90-MB), which achieved a 74.5% increase. This significant enhancement occurred when CFRP was applied to both sides of the web, covering both horizontal and inclined folds. In contrast, a similar specimen, L1200 (K90-HO), where CFRP was applied only to the horizontal folds on one side, exhibited a lower increase in shear load capacity, measuring 50.5%, which is almost 33% lower than the increase of shear load capacity with CFRP covering both horizontal and inclined folds of both sides of the web.

#### Effect of web slenderness on strength

**Fig. 16:**
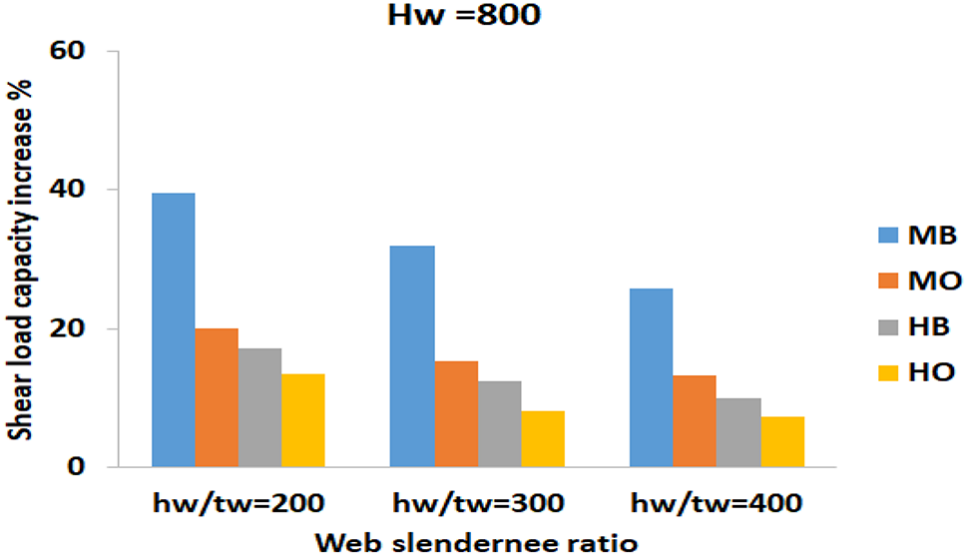
The effect of web slenderness for H_w_ = 800.

For main group H800 which is governed by interactive buckling, it is clear from the illustrated bar charts in Fig. 16, that the CFRP strengthening has the greatest effect on the beams with a low slenderness ratio (200), compared to the beams with a higher slenderness ratio (400). Within this main group, the maximum increase in shear load capacity was 39%, observed for specimen H800-200 (MB). In contrast, a similar specimen with a higher slenderness ratio, H800-400 (MB), exhibited a more modest increase in shear load capacity of only 25.9%.

## Proposed design procedure

**Fig. 17:**
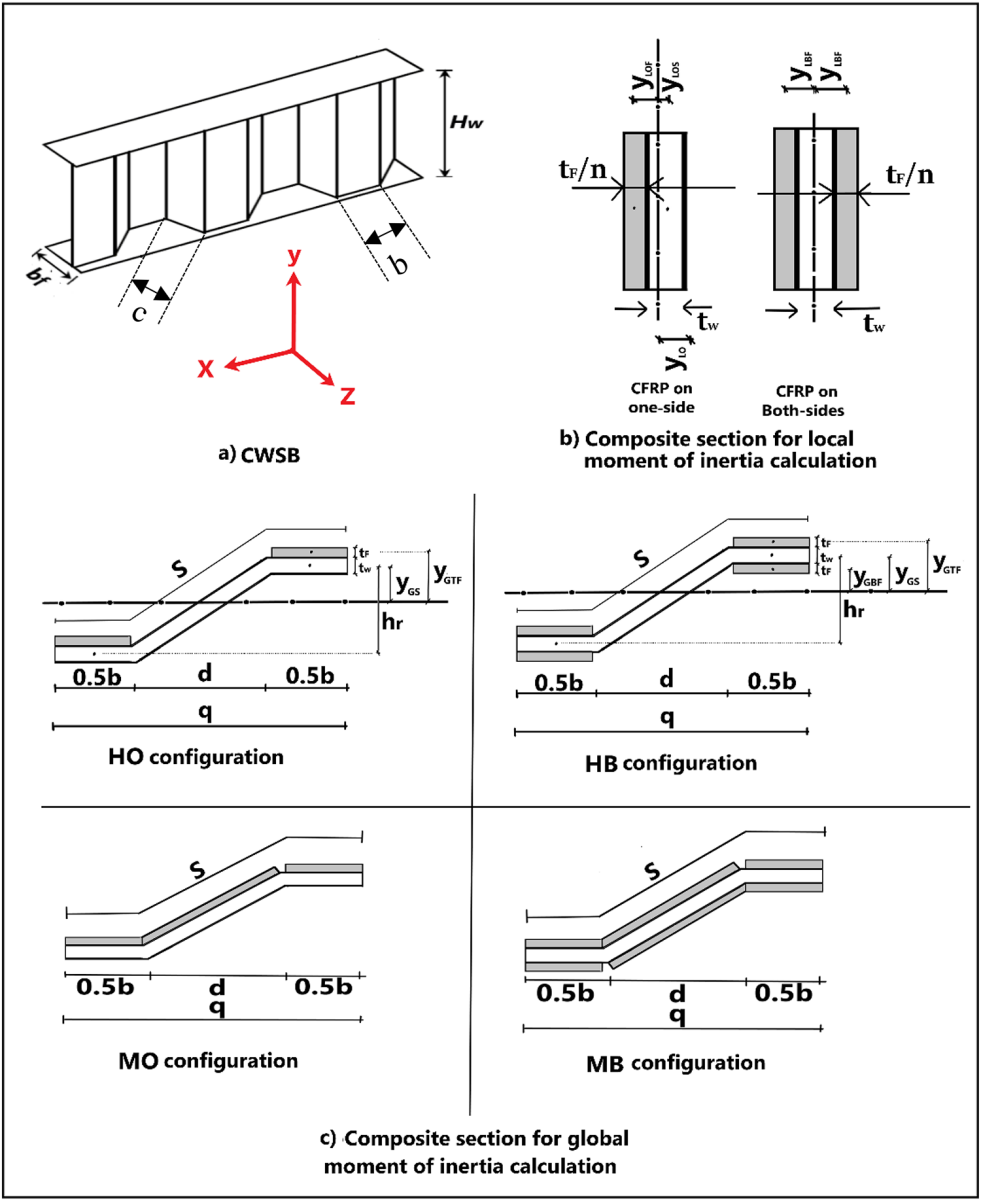
Definitions and geometric properties of strengthened CWSB.

In this section a proposed design procedure for predicting the design shear buckling strength of CWSBs strengthened by CFRP validated by finite element (FE) analyses is presented. This proposed procedure is a modified version of the previously published formulae of shear strength capacity of CWSBs by proposing to transform CFRP thickness into equivalent steel thickness by dividing the CFRP thickness by the modular ratio, n = E_s_/E_f_, where E_s_ and E_f_ are Young’s moduli of steel and CFRP respectively. Figure 17 illustrates the definitions and geometric properties of strengthened CWSB.

### Critical local shear buckling strength of CWSB

#### Critical local shear buckling strength of un-strengthened CWSB

The elastic local shear buckling stress of CWSBs, $\:{\tau\:}_{cr,L}$ can be determined by the classical plate buckling theory^13^ and expressed as:

5 $$\:{\tau\:}_{cr,L}=\:{K}_{L}{D}_{L}\:\frac{{\pi\:}^{2}}{{t}_{w}\:{w}^{2}}$$

Where:

*k*_*L*_, is local coefficient of buckling$\:=5.34+4{\left(\frac{w}{{H}_{w}}\right)}^{2}\:\:\:$for a practical design or 8.98 in case of clamped edge in case *t*_*f*_*/t*_*w*_ >3.

$\:{D}_{L}$ is the local flexural rigidity $\:=\:\frac{{E}_{s}\:}{1-{\nu}^{2}}\:{I}_{L}$, where $\:{I}_{L}\:\:\:$is the local moment of inertia

*E*_*s*_ is Young’s modulus of steel,

*ν* is Poisson’s ratio of steel,

*t*_*w*_ is web thickness.

*w* is maximum fold width = maximum of b and c which are shown in Fig. 17.

#### Critical local shear buckling strength of strengthened CWSB

The formula of $\:{\varvec{\tau\:}}_{\varvec{c}\varvec{r},\varvec{L}}$ mentioned before is used and being modified to suit the strengthened CWSBs.

For all configuration, CFRP in horizontal folds only, $\:{\tau\:}_{cr,L}$ is determined as follow:

6 $$\:{\tau\:}_{cr,L}=\:{K}_{L}{D}_{L}\:\frac{{\pi\:}^{2}}{{t}_{V}\:{w}^{2}}$$

Where: $\:{D}_{L}$ is the local flexural rigidity (all configuration) $\:=\:\frac{{E}_{s}\:}{1-{\nu}^{2}}\:{I}_{L}$, where, $\:{I}_{L}\:\:\:$is the local moment of inertia which determined for the composite section where, CFRP strip thickness is transformed into equivalent steel thickness by dividing it by the modular ratio, n.

$\:{t}_{V}$ is total thickness which is the equivalent sum of web thickness and CFRP thickness considering the difference in their elastic moduli ( $\:{t}_{w}+{t}_{F}/n$).

*w* is maximum fold width = maximum of b and c.

### Critical global shear buckling strength

#### Critical global shear buckling strength of un-strengthened CWSB

Elastic global shear buckling strength of CWSB, $\:{\tau\:}_{cr,G}$ is provided by^15^ as follows:

7 $$\:{\tau\:}_{cr,G}=\:{K}_{G}\:\frac{{D}_{x}^{3/4}{D}_{y}^{1/4}\:}{{t}_{w}\:{H}_{w}^{2}\:}$$

Where: D_x_ and D_y_ are the flexural rigidity about the x and y axes, respectively, $\:{K}_{G}$ is the coefficients of global shear buckling, K = 31.6 for simply supported, 59 for clamped.

#### Critical global shear buckling strength of strengthened CWSB

The formula of $\:{\varvec{\tau\:}}_{\varvec{c}\varvec{r},\varvec{G}}$ mentioned before is used and being modified to suit the strengthened CWSBs.

For all CFRP configuration, $\:{\tau\:}_{cr,G}$ is determined as follow:

8 $$\:{\tau\:}_{cr,G}=\:{K}_{G}\frac{{{D}_{x}^{3/4}D}_{y}^{1/4}}{{t}_{eqv.}\:{H}_{w}^{2}}$$

Where: $\:{t}_{eqv.}$ is the equivalent thickness of web and CFRP strip in one wave,

$\:{t}_{eqv.}=\frac{{(t}_{w}+{t}_{F}/n)\:b+{(t}_{w}+{t}_{F}/n)\:d}{q}\:\:\:\:\:$, q is the horizontal projection of half wave = b+d

$\:{D}_{x}$ is the longitudinal flexural rigidity about the x axes per unit length, $\:{D}_{x}=\frac{{E}_{s}}{q}\:\:{I}_{x}$, where $\:{I}_{x}$ is the global moment of inertia of strengthened CWSB about the x axes, $\:{I}_{x}$ is determined for the composite section for each configuration, based on transforming CFRP strip thickness into equivalent steel thickness by dividing it by the modular ratio.

$\:{D}_{y}$ is the transverse flexural rigidity about the y axes per unit length, $\:{D}_{y}=\:{E}_{s}\:{I}_{y,av}$ where, $\:{I}_{y,av.}$ is the average moment of inertia about the y axes for each CFRP configuration,

$\:{I}_{yav.}=\frac{{(I}_{y1\:}b)+{(I}_{y2}\:d)}{S}\:$, s is half wavelength = b + c

where, $\:{I}_{y1\:},{I}_{y2\:}$ are local moment of inertia of horizontal fold and inclined fold respectively, based on transforming CFRP strip thickness into equivalent steel thickness by dividing it by the modular ratio.

### Critical interactive shear buckling in CWSB

#### Critical interactive shear buckling strength of un-strengthened CWSB

$\:{\varvec{\tau\:}}_{\varvec{c}\varvec{r},\varvec{I}}$ developed by Bergfelt and Leiva-Aravena^30^ is presented as follows:

9 $$\:{\tau\:}_{Cr,I,n}=\frac{{\tau\:}_{Cr,L\:\:}{\tau\:}_{Cr,G}}{{({\tau\:}_{Cr,L}^{n}+{\tau\:}_{Cr,G}^{n})}^{1/n}}$$

Where, $\:{{\tau\:}_{cr,L}\:,\:\tau\:}_{cr,G}$ are the elastic local and global shear buckling stress of the un-strengthened CWSBs and the exponent n is selected to best fit the experimental data and several values are proposed for it^19,21,30^.

#### Critical interactive shear buckling strength of strengthened CWSB

The formula of $\:{\tau\:}_{cr,I}$ mentioned before is used and being modified to suit the strengthened CWSBs.

For all configuration, $\:{\tau\:}_{cr,I,r}$ is determined as follow:

10 $$\:{\tau\:}_{Cr,I,r}=\frac{{\tau\:}_{Cr,L\:\:}{\tau\:}_{Cr,G}}{{({\tau\:}_{Cr,L}^{r}+{\tau\:}_{Cr,G}^{r})}^{1/r}}$$

$\:{{\tau\:}_{cr,L}\:,\:\tau\:}_{cr,G}$ are the elastic local and global shear buckling stress of the strengthened CWSBs and the exponent r will be obtained in this study based on best fitting of the obtained results from the parametric study.

### Design shear buckling strength

#### Design shear buckling strength of un-strengthened CWSBs

Barakat and Leblouba^21^, investigated experimentally and analytically the shear strength of CWSBs and developed a model to estimate the normalized interactive shear buckling strength as a function of the interactive slenderness ratio, $\:{\lambda\:}_{I,n}$ which is presented as follow:

11 $$\:{\rho}_{S}=\frac{a}{{\lambda\:}_{I,n}^{m}}\:\le\:1$$

where, $\:{\lambda\:}_{I,n}$ is the shear buckling parameter which is function of critical interactive shear buckling strength $\:{\tau\:}_{cr,I,n}\:$ determined with *n* = 2 and defined as:

12 $$\:{\lambda\:}_{I,n}=\:\sqrt{\frac{{\tau\:}_{y}}{{\tau\:}_{cr,I,n}}\:\:}$$

a and m are constants to be determined, they used SOLVER in Excel^®^ which yielded the values a = 0.62 and m = 0.55.

#### Design shear buckling strength of strengthened CWSBs

The proposed normalized interactive shear buckling strength for strengthened CWSBs $\:{\rho}_{\varvec{F}}\:$is a function of design shear buckling strength of strengthened CWSBs, $\:{\varvec{\tau\:}}_{\varvec{F}}\:$and the yield shear strength, $\:{\varvec{\tau\:}}_{\varvec{y}}\:$which is presented as:

13 $$\:{\rho}_{F}=\frac{{\tau\:}_{F}}{{\tau\:}_{y}}$$

Based on the model developed by Barakat and Leblouba^21^, a new model to estimate $\:{\rho}_{\varvec{F}}$ is proposed by considering some variation in geometric properties and flexural rigidity for strengthened CWSB due to the presence of CFRP. The new proposed model for strengthened CWSB is based on full bond between CFRP and steel surface till failure.

In this model $\:{\rho}_{\varvec{F}}$ is a function of $\:{\varvec{\lambda\:}}_{\varvec{I},\varvec{r}}$ which presented as:

14 $$\:{\rho}_{F}=\frac{a}{{\lambda\:}_{I,r}^{m}}$$

Where, $\:{\lambda\:}_{I,r}$ is the shear buckling parameter which is function of critical interactive shear buckling strength of strengthened CWSBs.

Based on 105 FE results of strengthened CWSBs, a new model is developed to estimate the proposed normalized interactive shear buckling strength $\:{\rho}_{\varvec{F}}$ for strengthened CWSB. The new model is developed based on an optimization formulation with certain constraints obtained by analyzing the existing FE database and the prediction of existing models for the strengthened CWSBs. The target is to achieve the best difference percentage for proposed and FE normalized shear buckling strength ($\:\frac{{\rho}_{F}}{{\rho}_{FE}}$).

Accuracy of the prediction is assumed if the following condition is verified:

$$\:0.9\le\:\frac{{\rho\:}_{F}}{{\rho\:}_{FE}}\:\le\:1.0$$

In this optimization, the ratio of *t*_f_ /*t*_w_ ≥ 3.0 in all FE models, so a fixed boundary condition for web at juncture of the flanges is considered and the used value of local shear buckling coefficient (*K*_L_) is 8.98 and the used value of global shear buckling coefficient (*K*_G_) is 59. The optimization problem yielded the values of modified constants (a, m); a = 0.8 and m = 0.3 with *r* = 0.4 which achieved the best difference percentage of normalized shear buckling strength $\:\frac{{\rho}_{F}}{{\rho}_{FE}}\:\:$as follow:

$$\:Min.\frac{{\rho}_{F}}{{\rho}_{FE}}=0.93,\:\:\:Max.\frac{{\rho}_{F}}{{\rho}_{FE}}=1.15\:\:\:\:\:\:\:and\:\:Mean.\frac{{\rho}_{F}}{{\rho}_{FE}}=1.0$$

**Fig. 18:**
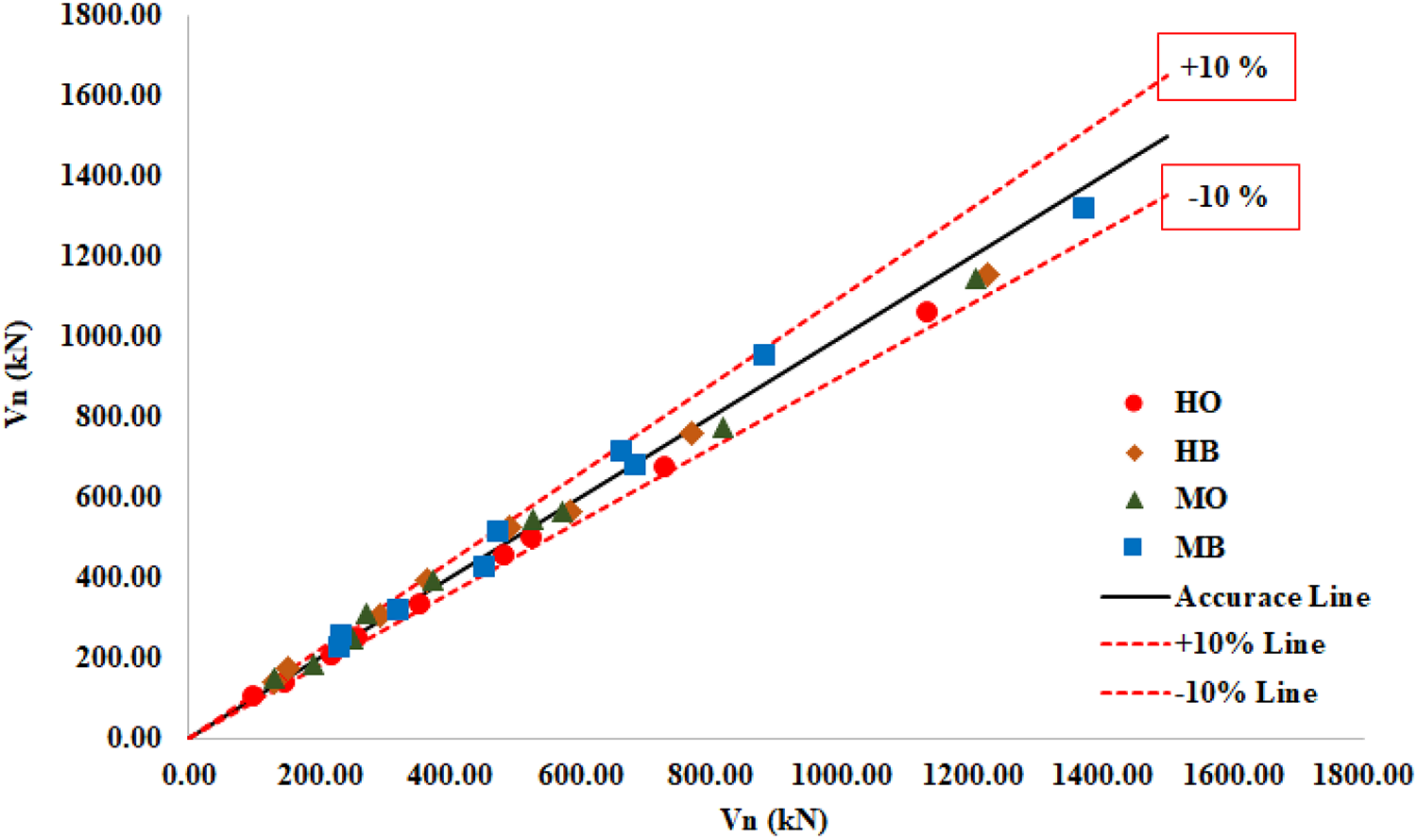
Comparison of the model prediction and FE results.

The comparison of the model prediction and FE results represented in proposed formulas load (V_n_) Versus FE load (V_FE_) is presented in Fig. 18.

### Results

- The narrow range of the maximum and minimum $\:\frac{{\rho}_{F}}{{\rho}_{FE}}\:$is 0.93, 1.15 respectively and the mean value is 1.0 indicate how well the proposed procedure performing for the whole FE database.
- The accuracy of the model has been verified to some extent from the results of $\:\frac{{\rho}_{F}}{{\rho}_{FE}}$, as it is found that 59% of the results for $\:\frac{{\rho}_{F}}{{\rho}_{FE}}$ is in range between (0.9–1.0), while 41% of the results is in range between (1.0–1.15)$\:,\:$and 0% of the results is for $\:\frac{{\rho}_{F}}{{\rho}_{FE}}<0.9$.

### Limitations and applicability of the proposed design procedure

The proposed design procedure for corrugated web steel beams (CWSB) strengthened with CFRP strips should be applied with caution within the following limitations, which were considered during its development:

- The steel material should be limited to A36 to ensure consistency with the steel grade used in the parametric study.
- The adhesive type and CFRP strips should exhibit similar mechanical properties to those utilized in the current research.
- The web depth should range between 500 and 1200 mm, corresponding to the range covered in the parametric study.
- The web slenderness ratio (H_w_/t_w_) should be between 200 and 400, while the corrugation flat width to web height ratio (b/H_w_) should range from 0.06 to 0.26.
- A suitable strength reduction factor should be applied in any final design proposal to account for an average overestimation of approximately 6.8% in the FE model results, which served as the basis for the parametric study and the development of the proposed design procedure.

## Conclusion

This paper presented numerical investigations on CWSBs strengthened with CFRP to assess the effectiveness of applying CFRP as a strengthening technique to the CWSBs in improvement the shear capacity and to develop an empirical formula to predict the design shear buckling strength of strengthened CWSBs. In this study, (FE) model was developed in ANSYS software which accurately verified using the previous experimental results. Parametric studies are conducted using the validated FE model involving 105 CWSBs to investigate the influence of different parameters not included in tests. Finally, a design procedure is established to predict the design shear buckling strength of strengthened CWSBs.

The following conclusions can be drawn:

1. The results of the FEA demonstrated the efficiency of using CFRP strengthening technique in enhancement the shear capacity of CWSBs up to 74.50%.
2. Increasing in the shear capacity is strongly depending on CFRP configuration, it is highly recommended to apply CFRP strips on horizontal and inclined folds on both-fold sides (MB) as the increase in shear load capacity can be as much as 33% of the similar cases with CFRP applied to horizontal folds one side of the web.
3. The effects of different parameters on strength have been presented using bar charts as follow

- The increase in CFRP length does not result in a substantial improvement in beam capacity, as a CFRP strip length to web height ratio of 70% has already achieved approximately 93% of the shear load capacity observed in specimens with a CFRP strip length to web height ratio of 90%. Changing CFRP thickness from 1.2 to 1.4 mm has an insignificant effect on beam capacity increasing. The maximum observed difference in the increase of shear load capacity between CFRP thickness 1.2 and 1.4 mm within the studied specimens was 4%.
- The CFRP strengthening has the greatest effect on the beams with a local buckling mode (L), compared to the beams with a global buckling (G). It is observed that specimens with global buckling failure mode can achieve as low as 73.2% of the shear capacity increase of similar specimen with local buckling failure mode.
- Among all CFRP configurations, applying CFRP on horizontal and inclined folds on both-fold sides has the largest strength gain increasing which could result in increase in shear load capacity as high as 50% of that for case of applying CFRP on horizontal folds on one side of the web.

4 A proposed formula for predicting the design shear buckling strength of CWSBs strengthened with CFRP is presented. The comparison of the proposed model prediction and FE results showed a good consistency indicated the accuracy of the proposed formula.

## Data Availability

Correspondence and requests for materials should be addressed to Sherif M. Ibrahim.
